# Separating Teeth

**Published:** 1870-12

**Authors:** J. N. Crouse


### ARTICLE III.

### *Separating Teeth.*

By Dr. J. N. Crouse.

Read before the Illinois State Dental Society.

The answer to this question, "When should teeth be separated?" divides itself naturally into three divisions, viz : to prevent decay ; to make a correct diagnosis ; and to get room when they have decayed to fill them.

Until recently the dental profession was so strongly prejudiced against the practice of filing or cutting the teeth apart, on the ground that removing the enamel caused the teeth to decay ; that through their instrumentality and the seeming reasonableness of this theory, a very strong prejudice exists in the minds of the people and some of the profession, against filing the teeth. Yet observation has taught us the incorrectness of the opinion that filing teeth causes them to decay, and on the other hand, we are fully persuaded that in many cases much good results from separating the teeth, thereby preventing them from decaying. Now I have no idea of advancing the doctrine that teeth of children, or quite young patients, should be separated by filing them apart when decay has not commenced, or if superficial decay is found in the approximal surface, unless it is quite superficial, and can be removed without making much of a separation. I should prefer to fill rather than remove by filing them apart. Although much expense and trouble might be saved by separating the front teeth, freely, when decayed, of even young persons, leaving the space between them wider at the cutting edge than at their necks, and thus removing superficial decay, yet at this period of life, when the teeth are most subject to decay, and when beauty is most valued, especially by the female sex, the advantage of such treatment is often more than counterbalanced by the mental suffering which the patient would experience by destroying the beauty and symmetry of these organs. Hence duty demands that we should not destroy their beauty at an age, when it is most valued, but repair the loss, when decay takes place, by filling; thus remedying the evil when it comes, rather than prevent it from coming. But in adults, when the teeth come together at the line of the grinding and cutting surfaces, leaving a space at the neck, making a repository for food, which is exceedingly difficult to remove, I should, in a majority of cases, separate them with a file, employing chisels when they can be applied to advantage; not merely cutting them apart so that the space at the edge and necks is equal, but restoring the convexity of the surface, being careful to polish out all the scratches, and leave the surface as smooth, if possible, as well formed enamel.

I do not advocate the separating of the teeth indiscriminately where they are together at the edges and space toward the gums. If teeth, in such a condition, can be readily cleansed of the food which lodges in this space, and the patient will exert sufficient care in keeping them thoroughly cleansed, and if decay has not commenced, I should not interfere. But we often see teeth in such a shape and position that it is next to impossible for the patient to remove the deposits from about them.

This is often the case when the gums and alveolar processes have receded and left the necks exposed. By making a careful examination we will find decay to a greater or less extent in a large majority of cases where we have the conditions refered to, especially if they are of long standing. Hence, in all such cases, I should recommend separating them freely in the manner already alluded to. Especially when we have this condition of the gums and alveola, the necks of the teeth being exposed to the action of corrosive agents, would make thorough work of separating; for when these teeth decay, it is usually upon the necks, and of such a nature that it is very unsatisfactory filling them, as the decay extends over so much surface and crops out here and there, so that there are no well defined margins. Consequently, I prefer prevention rather than cure.

*Second.* Teeth should be separated for the purpose of making a correct diagnosis. That is, when we have the care of teeth which are crowded together so that we cannot ascertain *beyond a doubt* whether decay has or has not taken place, we should invariably separate them sufficiently to make a correct diagnosis. This is best done with pressure, either by driving a wedge with sufficint force to move them enough to ascertain their condition, or by separating them by a gradual pressure, which can be done by packing cotton, or cotton and sandarach, between them; or if they are much crowded, by drawing a thread or floss silk between them until sufficient space is obtained to insert a pine wedge. In this way we can separate several teeth at the same time if it is desired. We should continue this until sufficient room is obtained to determine whether or not decay has commenced on the approximal surfaces.

*Thirdly.* Separating to obtain space when we wish to fill the approximal cavities in cases where we have not ample space without it. How is it best done? This is a qnestion in regard to which much difference of opinion exists, some advocating separation with file and chisel; some by rapid wedging in all cases, while others prefer to move them apart by gradual pressure, employing cotton and sandarach varnish, rubber or pine wood, each claiming his method to be the best one. I shall not denounce either of these modes, but will make an effort to show where each can be used to advantage. In the back teeth, where filing them apart will not mar their beauty, and where less space is required from the fact that in the great majority of cases, where these teeth are decayed on the approximal surfaces, thoroughness requires that we cut from the grinding surface to the cavity in the approximal surface, thereby securing space, unless this approximal cavity is very small, in which case, it would be proper first to use a gradual pressure till space enough can be had, with what filing we wish to do, to perfect the operation.

The incisors and cuspids should generally be separated by pressure. And at this point I wish to enter my protest against the practice of separating indiscriminately by rapid wedging. Now and then, we meet with cases where this method possesses some advantages. Where the teeth have lost part of their sockets and are loose, where time cannot be afforded to separate by gradual pressure, and the patient is of such a temperament, or the parts surrounding the teeth are of such a character, that to force them apart with a wedge causes but little pain, we may pursue this method, if a proper amount of care is observed in the operation.

Yet, in a large majority of cases, to drive the teeth apart with a wooden wedge and mallet, is, in my opinion, a barbarous practice, causing unnecessary pain, sometimes exfoliation of the alveolar process, and often endangering the life of the pulp. Hence, in most cases I think that the preferable way is to separate by gradual pressure. For this purpose, sandarach varnish and cotton is very good, especially between the molars where it is difficnlt to insert a pine wedge. As to rubber, I do not consider it a proper material for this purpose, as the elasticity is so great, that as long as it has any amount of bearing it exerts sufficient force to move the teeth, and especially is this an objection when the teeth are sore, which causes them to move more easily than if not, and increases the soreness in a corresponding degree. In most cases, therefore, I should separate by the use of pine wedges, pressing them between the teeth with sufficient force to gradually move them apart so as to accomplish the purpose without producing any great degree of sensitiveness; and should the teeth be found too sensitive to pressure after the space has been secured, I should leave them for a short time until the inflammation has subsided, retaining the space already gained.

